# Complex Sex Determination in the Grey Mullet *Mugil cephalus* Suggested by Individual Whole Genome Sequence Data

**DOI:** 10.3390/ani15162445

**Published:** 2025-08-20

**Authors:** Mbarsid Racaku, Serena Ferraresso, Massimiliano Babbucci, Andres Blanco, Costas S. Tsigenopoulos, Tereza Manousaki, Jelena Radojicic, Vasileios Papadogiannis, Paulino Martínez, Luca Bargelloni, Tomaso Patarnello

**Affiliations:** 1 Department of Comparative Biomedicine and Food Science, University of Padova, 35020 Legnaro, Padua, Italyluca.bargelloni@unipd.it (L.B.); tomaso.patarnello@unipd.it (T.P.); 2 Department of Zoology, Genetics and Physical Anthropology, Faculty of Veterinary, Universidade de Santiago de Compostela, Campus Terra, 27002 Lugo, Spainpaulino.martinez@usc.es (P.M.); 3 Institute of Marine Biology, Biotechnology and Aquaculture, Hellenic Centre for Marine Research, P.O. Box 2214, 71003 Heraklion, Crete, Greece; tsigeno@hcmr.gr (C.S.T.);

**Keywords:** *Mugil cephalus*, sex determination, sex-associated markers, genome assembly, intraspecific variation

## Abstract

*Mugil cephalus* is a cosmopolitan marine fish highly relevant from ecological and economic perspectives. Previous studies identified sex-associated variants in the follicle-stimulating hormone receptor (*fshr*) gene following an XX/XY sex determination (SD) system. However, these variants could not be fully associated with sex in all samples. This suggests other genes and/or environmental factors may be involved in the SD of this species, denoting intraspecific variation. In this study, we constructed a new high-quality genome assembly of *M. cephalus*. We then re-sequenced the whole genome in males and females from two divergent Mediterranean populations to ascertain whether other genetic variants could also be involved in SD. *fshr* gene variants showed to only partially explain SD, while a new intronic variant in the *sestd1* gene appeared to be associated with SD following a ZZ/ZW system. The presence of other putative candidate SD variants showing significant differences between the two populations suggested a regional pattern of variation in SD in the Mediterranean Sea. The incomplete association of all the identified variants also pointed to a potential role for environmental factors.

## Simple Summary

Determining whether a fish will develop as male or female is a crucial issue for aquaculture, as it can significantly impact growth rates, reproduction, and production planning. For the flathead grey mullet (*Mugil cephalus*), a commercially valuable fish, the genetic factors that control sex determination are still unclear. In this study, we generated a new genome assembly and compared genomic data of male and female fish from two distinct Mediterranean populations (Aegean and Tyrrhenian Seas). We found that a previously known sex-related gene was relevant in the Tyrrhenian population but not the Aegean one, and we also identified other putative sex-associated variants which might play a role in sex determination. These results suggest that sex in the flathead grey mullet is influenced by several genetic factors and probably also by the environment. Our works provides new insight for the better understating and management of reproduction in this species.

## 1. Introduction

The flathead grey mullet, *Mugil cephalus*, is a teleost belonging to the family Mugilidae, which comprises 25 genera and 76 species [1]. It is a cosmopolitan marine fish highly significant both from ecological and economic perspectives. *M. cephalus* has been a mainstay of traditional aquaculture for centuries, especially in the Mediterranean region. The traditional Italian delicacy “bottarga” is the primary factor contributing to the commercial relevance of *M. cephalus*. This is a product made from salted and dried female gonads that is considered a luxury food (the price is more than 200 EUR/kg) in numerous regions of the world [2]. Mullet roe is a costly product used to supplement fish flesh, particularly in Asian and Mediterranean markets [3].

The flathead grey mullet is a species without obvious sexual dimorphism, although females grow slightly faster than males and reach bigger sizes. Its aquaculture production increased from 25,600 tonnes in 1997 to 147,000 tonnes in 2003 [4], with growing interest also motivated by mullets being a low-trophic species. Furthermore, different studies consider *M. cephalus* an excellent sentinel for environmental health, particularly for hypoxia and heavy metal pollution [5]. High levels of heavy metal pollution have been found in the gonads of this species but also in fish organs where metals accumulate during crucial stages of embryonic development, negatively affecting embryogenesis [6].

Teleost fish exhibit remarkable diversity in physiology, behaviour, and developmental processes, including sex determination mechanisms, which show huge variation across the different taxonomic levels [7,8]. Unlike the heteromorphic and highly differentiated sex chromosomes of mammals and birds, the sex chromosome pair of most fishes is homomorphic and slightly differentiated [9]. Indeed, only around 5–10% of fish species was shown to possess heteromorphic sex chromosomes [10,11]. In fish, sex chromosomes have undergone quick evolutionary changes characterised by transitions between SD master genes (Master SD, MSD, genes) and different SD systems [12].

Sex determination in teleosts can range from strictly genetic sex determination (GSD) to environmental sex determination (ESD) mechanisms [13]. This plasticity may allow for rapid adaptation to changing environmental conditions, contributing to the evolutionary success of teleosts [14], as demonstrated by the nearly 30,000 species described for this taxonomic group accounting for half of all extant vertebrates and approximately 98% of all ray-finned fish species. While several genetic loci underlying SD have been reported, environmental factors such as temperature, photoperiod, social interaction, pH, and hypoxia also play a significant role in many species [15]. Within GSD systems, fish display diverse mechanisms including male heterogamety (XY system), female heterogamety (ZW system), and polygenic SD with or without multiple sex chromosomes [12,16].

The increasing information on SD mechanism in teleosts has demonstrated a rapid evolutionary turnover, divergence, and diversity, even between closely related species [17]. The genetic architecture of SD in fish often resembles a complex trait, involving major and minor genetic factors, environmental influence, and potential epistatic interactions between genes [12]. GSD can range from fully heteromorphic chromosomes to cryptic differences that are only detectable at the molecular level. The same SD genes have been recruited repeatedly along evolution, mostly belonging to specific developmental pathways. These include transcription factors at the top of the sex differentiation cascade, the transforming growth factor beta (TGF-β) pathway and, more recently, genes implicated in the steroidogenesis pathway. Up to now, more than 21 distinct SD genes have been found across 114 fish species spanning 18 orders, most of which are connected to these particular pathways [17].

Previous studies on *M. cephalus* identified several single-nucleotide polymorphisms (SNP) associated with sex in the follicle-stimulating hormone receptor *(fshr)* gene [18,19]. Subsequently, the *fshr* gene was also reported in *Solea senegalensis* as the MSD gene following an XX/XY SD system [20]. Ferraresso et al. [18] identified three *fshr* SNP variants associated with sex in exon 14 (Muce179, Muce206, and Muce322), the first two corresponding to missense mutations and the third one to a synonymous variant. Later, Curzon et al. [19] and Anderson et al. [21] reported *fshr* SNP variants in both exon 1 and 14; those in exon 14 (c.1732G>A and c.1759T>G) correspond to the Muce179 and Muce206 variants from Ferraresso et al. [18].

However, these two main variants were not completely associated with phenotypic sex in all samples. Ferraresso et al. [18] studied two Mediterranean regions, the Aegean and the Tyrrhenian, and revealed variable associations of *fshr* genotypes with sex, especially for males. In fact, in the Aegean region the association between phenotype and genotype at *fshr* in males was around 50% (heterozygous WT/mut1: wild type/alternative variant), while in the Tyrrhenian region, it was approximately 86%, very close to the 90% reported in an Israeli Mediterranean estuary by Curzon et al. [19]. These observations suggest that other genes and/or environmental factors may be involved in the SD of this species, denoting intraspecific SD variation. Intraspecific SD variation has been documented in different fish species, such as Northern pike *(Esox lucius)* or Atlantic silverside (*Menidia menidia*) [22].

In Ferraresso et al. [18], a pool-sequencing approach was used on a Tyrrhenian population sample to identify putative SD loci against a draft mullet genome. Nucleotide variants at the most significant locus, *fshr*, were tested using targeted amplicon sequencing on the Tyrrhenian individual samples and a second population from the Aegean Sea. In this study, we assembled the whole genome of grey mullet to provide a robust genomic framework to identify genetic variants putatively associated association with sex. A subset of samples from the two populations analysed in Ferraresso et al. [18] was re-sequenced at the whole genome level to assess whether other genetic loci in addition to *fshr* might be involved, thus providing a more comprehensive explanation for SD in the species.

## 2. Materials and Methods

### 2.1. Genome Assembly

#### 2.1.1. DNA Extraction and Nanopore Sequencing

One juvenile specimen of flathead grey mullet (*M. cephalus*), captured in December 2020, was used for genome assembly. DNA extraction were performed using Miller salt extraction [23] with RNase digestion and chloroform steps added, followed by final elution in 5 mM TRIS/HCl pH 8.5 buffer. This DNA was further purified using QIAGEN Genomic-tip 20/G (Cat. No.: 10223, QIAGEN, Hilden, Germany), with elution in 55–60 µL TE. The purification process involved two rounds of pooling ten separate DNA extractions, resulting in approximately 40 µg of DNA for genome sequencing and assembly.

After genomic tip purification, the DNA was size-selected following centrifugation with PEG8000 and NaCl, which depletes DNA below 4–6 Kb. A total amount of 12.4 µg of HMW DNA was finally obtained as measured in a Qubit fluorometer and NanoDrop microvolume spectrophotometer.

Four LSK109 libraries were constructed using the Ligation sequencing kit (SQK-LSK109) and deployed across five 5 flow cells on the MinION and MinION Mk1C portable sequencers (Oxford Nanopore Technologies, Oxford, UK). The end-prep step was extended for 35 min, enhancing the proportion of longer reads rendering a coverage of 142×. MinION and the MinION Mk1C were operated in parallel and displayed comparable performance. Raw sequencing data from Illumina sequencing yielded an average mean depth ~48×.

#### 2.1.2. Genome Assembly

For genome assembly, a combination of bioinformatic tools was employed to process and analyse both short and long-read sequencing data. Trimming for Illumina reads was performed with Trimmomatic v0.39 [24], and for MinION reads with Porechop v0.2.4 [25]. Quality control was performed with FastQC v0.11.8 [26] for Illumina data, and Nanoplot v1.33.0 [27] for MinION data, ensuring the integrity of input sequences.

The initial assembly was performed with long-read data using the Flye assembler v2.8.1 [28] with the parameter “--scaffold” for self-scaffolding, which is specifically designed to handle these data efficiently. To improve the accuracy of this initial assembly, we implemented two steps of the polishing process. First, we used Racon v1.4.3 [29] and Medaka v1.6.1 [30] to polish the long-read assembly, leveraging the length and coverage of these reads. Subsequently, further refinement of the assembly was performed with Pilon v1.24 [31], which incorporates the high accuracy of short-read data to polish errors.

To assess the quality of our final assembly, we employed QUAST v5.0.2 [32] for evaluating contiguity metrics and BUSCO 5.4.7 [33] for assessing completeness, providing a comprehensive evaluation of our genome assembly.

The mitochondrial DNA genome was retrieved from the assembly using GetOrganelle v1.7.6.1 [34] and its annotation was performed with MITOS v2.1.9 [35]. This genome was removed for further analysis.

#### 2.1.3. Genome Annotation

Annotation was performed previously masking the genome following the user guide of FasTE [36]. First, a transposable element (TE) library was constructed using EDTA [37] for their annotation in the target genome. Using the --sensitive parameter, EDTA uses RepeatModeler v2.0.1 [38] to identify remaining TEs.

Subsequently, the EDTA library was classified with DeepTE.py [39], to be used as an input for RepeatMasker [38], which employs a library of TEs to filter repetitive DNA sequences, masking them. The library used for genome masking was a combination of the de novo TE library created from the genome of *M. cephalus* and FishTEDB [40], a fish-specific TE library.

De novo gene prediction was performed using the soft-masked genome with MAKER v3.01.03 [41] pipeline. First, an ab initio gene prediction was performed using SNAP [42], GeneMark-ES v4.70 [43], and Augustus [44]. Genome functional annotation was performed from BLAST+ v 2.9.0 [45] queries of extracted protein sequences against Unitprot [46] and the InterPro database [47]. The final GFF produced was analysed with AGAT v1.0.0 [48] to fix, check, and add missing information to create a complete, sorted, and standardised gff3 format.

#### 2.1.4. Alignment of *M. cephalus* Genome Assemblies

*M. cephalus* genome assembly was mapped against the chromosome-level genome assembly of *M. cephalus* constructed from a Chennai Indian sample (CIBA_Mcephalus_1.1, GenBank GCA_022458985.1) [49] using minimap2 v2.25-r1173 [50]. For comparative analysis, 155 contigs and 26 scaffolds, representing ~91.2% of our total genome assembly, were homologous to the CIBA_Mcephalus_1.1 reference genome, indicating the high genomic correspondence between them (Table S1). Despite the high similarity between the two genomes, we decided to employ the genome assembly generated in the present study for SNP calling, considering the genetic differentiation that might exist between Indian and Mediterranean populations, as also documented in a recent work by Thieme and colleagues [51].

### 2.2. Whole Genome Sequencing (WGS) SNP Genotyping

#### 2.2.1. Sample Collection and Genotyping

**Figure 1:**
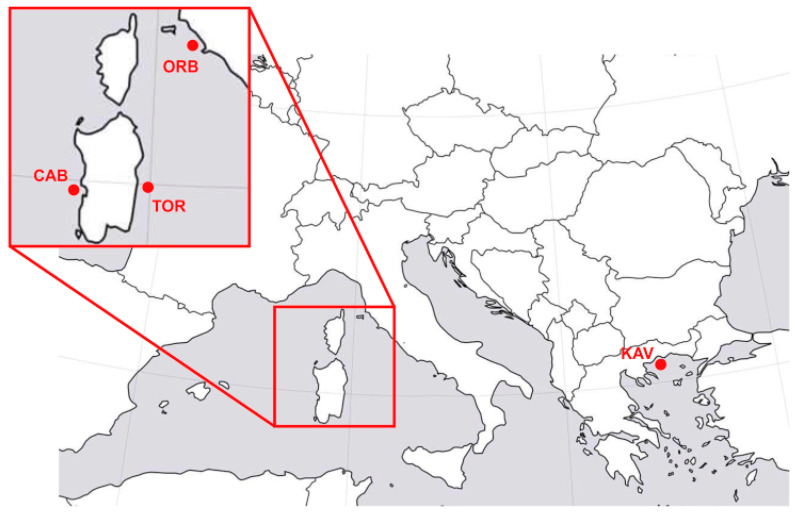
Sampling sites of *M. cephalus* (ORB: Orbetello Lagoon; CAB: Cabras Lagoon; TOR: Tortolì Lagoon; KAV: Kavala) from Ferraresso et al. [18].

The *M. cephalus* samples came from two distinct Mediterranean areas, the Aegean and the Tyrrhenian (West Mediterranean) Seas (Figure 1). The Aegean samples were collected in Kavala (Northern Greece), whereas in the Tyrrhenian Sea, samples were collected at Cabras, Orbetello, and Tortolì (Italy).

The sampling, DNA extraction, and *fshr* genotyping of these samples were previously conducted [18]. From this dataset, we selected a subset of individuals for whole genome sequencing intending to explore other genomic regions putatively involved in SD that could explain the lack of full association of *fshr* with sex in the species.

#### 2.2.2. Sample Sequencing

Genomic libraries for paired-end whole genome sequencing (WGS) were prepared according to Illumina DNA Prep Tagmentation (cat#20018704, San Diego, CA, USA) protocol. The quality of the libraries was assessed using Agilent 2100 Bioanalyzer (Palo Alto, CA, USA) and each DNA library was quantified using the Qubit^®^ dsDNA HS Assay Kit (Invitrogen, Carlsbad, CA, USA). Sequencing was performed on the Novaseq6000 machine (Oslo, Norway) with an average mean depth ~28× per sample.

A total of 72 individuals were selected for WGS, namely 32 females and 40 males. The criterion for sample selection from Ferraresso et al. [18] was to include individuals with expected and unexpected genotypes according to the *fshr* information to identify other putative loci underlying SD in *M. cephalus*. Thus, for both geographic regions, 16 females were selected, with 12 of them showing the expected WT/WT *fshr* genotype, but 4 showing WT/mut1. Additionally, of the 20 males from each population, 11 were had the WT/mut1 genotype and 9 had the unexpected WT/WT (Table S2).

#### 2.2.3. Raw Reads Processing and SNP Calling

Raw sequencing data from Illumina sequencing were checked for quality using FastQC software v0.11.5. Then, raw reads were trimmed with Trim Galore! v0.6.10 [52] to remove adaptors and low-quality bases; default parameters were applied except for length (set to 70) and q (25), and the option “nextera” was applied to remove adaptors.

The trimmed reads were mapped against our reference genome using the BWA version 0.7.17-r1188 [53]. To ensure that subsequent analyses with PicardTools [54] and variant calling were compatible, the -c 1 and -M parameters were used. Every SAM file produced from BWA was converted and sorted into a BAM file using SAMtools [55]. Finally, we identified and removed any duplicate reads with PicardTools v3.0.0 using default mode.

The reference genome and the final read alignment were used as input for the variant calling BCFtools v1.17 [55] using mpileup, call, and filter commands. To avoid keeping alignments with low mapQ, we set -q to 20 for the mpileup command and the -m parameter for call. Filtering was performed with BCFtools to remove indels setting -g to 5. Finally, VCFtools v0.1.16 [56] was used to retain only bi-allelic sites with minor allele frequency (MAF) > 0.15 and to remove sites that contained any indel or missing data. The same workflow for mapping and SNP calling was performed using the CIBA_Mcephalus_1.1 genome [49].

The VCF data contained genetic information from 1275 contigs and 29 scaffolds. To refine the analysis, the CIBA_Mcephalus_1.1 genome was used, selecting the 155 contigs and 26 scaffolds outlined before. Subsequently, the main VCF file was reorganised by grouping these selected contigs into the 23 reconstructed chromosome VCF file. This approach allowed to anchor the genetic data to the chromosomes of the species, aiding meaningful genomic analyses.

### 2.3. Sex Determination (SD) in M. cephalus

#### 2.3.1. Genetic Differentiation

Since no significant genetic differentiation was detected among the three Tyrrhenian locations studied (see Section 3), they were grouped in a single population. Genetic differentiation between the two main geographical areas studied (Tyrrhenian and Aegean) was performed as a necessary reference for further analyses on intraspecific SD variation in *M. cephalus*. The relative component of genetic differentiation (F_ST_) was estimated with Genepop 4.7 [57]. A discriminant analysis of principal components (DAPC) was also performed for an integral visualisation of genetic differentiation using the two most informative components [58]. Based on this information, the Tyrrhenian and Aegean samples were analysed as two differentiated regions (see Section 3). Then, the analysis with VCFtools v0.1.16 was repeated starting from raw data to avoid losing any region-specific genetic variants.

#### 2.3.2. Identification of the SD Regions

Each reconstructed chromosome was screened to estimate the relative component of genetic differentiation between males and females (F_ST_) and the intrapopulation (sex) fixation index (F_IS_) [59] using adegenet packages v2.1.10 [60] from each vcf chromosome file. F_ST_ and F_IS_ were explored to identify genomic regions associated with sex, consistent with a ZZ/ZW or XX/XY SD system (F_ST_ = 0.5; F_IS_ = −1), reflecting allele fixation in the homogametic sex and full heterozygosity in the heterogametic sex. Candidate SNPs with associations were also selected based on their associated *p*-values (*p* < 0.005), calculated with Genepop 4.7 [57].

## 3. Results

### 3.1. Genome Sequencing

The Oxford Nanopore Technologies (ONT) MinION sequencing of the juvenile *M. cephalus* used for genome assembly produced 13,613,901 raw reads, amounting to a total of 100,081,010,194 bp with an N50 of 13,358 bp. After trimming, the MinION data resulted in a slightly increased read count of 13,626,708, which can be attributed to the computational splitting of reads containing internal adapters, thus comprehending 99,527,883,358 bp and very similar N50. All data suggested high-quality sequencing with minimal adapter contamination or low-quality regions. Illumina 150 bp pair-end (PE) sequencing of the same individual generated 126,368,599 raw reads, 109,184,810 of them being retained after trimming.

### 3.2. Genome Assembly and Annotation

The final assembly consisted of 1275 contigs and 29 scaffolds, a total length of 658,521,108 bp, and a contig N50 of 8.38 Mb with a maximum contig length of 30.5 Mb. The completeness results from BUSCO, using the Actinopterygii database, yielded 98.2%, with 97.6% single-copy and 0.6% duplicates. The identification of transposable element (TE) resulted in 31.9% of the total ONT-generated genome (~211 Mb) (Table S3) within the range reported for other teleost species (1.6% to 37.1%) [61].

After masking TEs in the genome assembly, a total of 26,536 protein-coding genes were predicted from the MAKER pipeline. The BUSCO completeness of gene annotation using the same Actinopterygii database was 92.1%, with 90.7% single-copy and 1.4% duplicates.

The mitochondrial genome was obtained as a single contig encompassing 16,706 bp and including 37 annotated coding genes (Figure S1).

### 3.3. SNP Calling

The average number of 150 bp PE Illumina reads per sample was 74.2 million, with 72.7 million being retained after trimming. The raw read count per sample ranged from a minimum of 56.7 million (54.7 million after trimming) to a maximum of 104.6 million (102.2 million after trimming) (Table S4). The percentage of mapped reads in the reference genome was 99%. BCFtools identified a total of 2,835,032 SNPs after filtering (MAF > 0.15) from the total number of aligned reads from the 72 samples against our genome assembly.

### 3.4. Comparison CIBA_Mcephalus_1.1

The alignment between the CIBA_Mcephalus_1.1 genome against our genome assembly rendered 99.8% sequence homology. Although the BUSCO metrics of our assembly were similar to CIBA_Mcephalus_1.1, the contiguity was significantly lower. By selecting only the 155 contigs and 26 scaffolds that matched the CIBA_Mcephalus_1.1, the contiguity of our genome assembly was improved (Table S5). However, a portion of some contigs were found to be present on both chromosome 2 and the very small chromosome 24. We decided to maintain those contigs on the bigger chromosome 2.

We compared the number of SNPs identified in our 72 individuals using the two reference genomes. This analysis revealed 4,813,525 SNPs with the CIBA_Mcephalus_1.1 genome, significantly higher than the 2,835,021 SNPs identified when using present study’s genome assembly as the reference. The increase in the number of SNPs detected (58.9%) might be explained by the high geographical distance between the populations to which the individuals used for assembly belong, and also by the higher completeness of the CIBA_Mcephalus_1.1 genome. However, considering the genetic divergence between the Mediterranean Sea and the Indian Ocean samples, we opted to use our assembly as a reference for SNP calling and genotyping.

### 3.5. Genetic Structure

**Figure 2:**
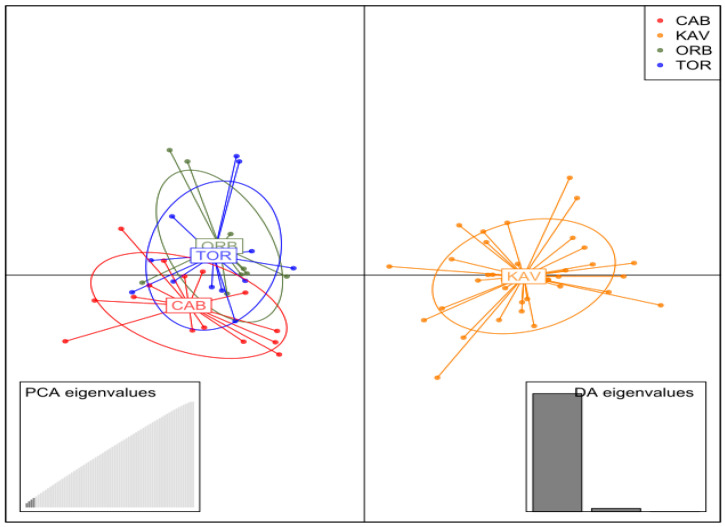
Scatterplot of DAPC analysis in the *M. cephalus* using 2,835,032 SNPs. Clusters and inertia ellipses for each sample are shown in different colours (ORB: Orbetello; CAB: Cabras; TOR: Tortolì Lagoon; KAV: Kavala).

Genetic differentiation between Tyrrhenian samples was low and not significant (*p* > 0.05), thus they were subsequently treated as a single population (Figure S2). Considering the low but significant genetic differentiation between Tyrrhenian and Aegean regions (F_ST_ = 0.0041, *p* < 0.05; Figure 2), we performed SNP calling within each region to avoid losing genetic variants exceeding the MAF threshold only in one region.

Following the outlined procedure, 2,814,512 SNPs were called in the Aegean population, and 3,013,979 SNPs in the Tyrrhenian one. We should note that the reference genome was assembled using an individual belonging to the Aegean region. Additionally, the main 155 contigs and 26 scaffolds previously selected which matched the CIBA_Mcephalus_1.1 genome were subsequently extracted from the VCF file, and the number of SNPs retained was similar to those observed in the original VCF file. A total of 2,871,004 SNPs were called for the Tyrrhenian population, and 2,676,984 SNPs were called for the Aegean population.

### 3.6. Screening the Genome for Sex-Associated SNPs

The number of SNPs examined for genome screening in the Aegean and Tyrrhenian populations ranged from 65,293 and 69,320 SNPs in C23 to 217,950 and 231,210 in C1, respectively (Table S6).

**Figure 3:**
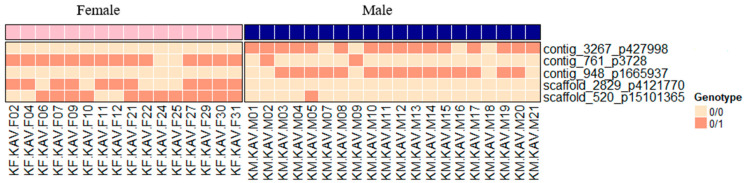
Diagram showing the allele frequency of the allele of the heterogametic sex (Y or W) for the four SNPs meeting the sex-association criteria (F_IS_ < −0.5 and F_ST_ > 0.3) in the Aegean population.

**Figure 4:**
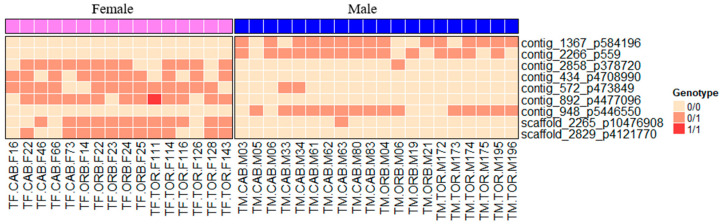
Diagram showing the genotypes of males and females for the SNPs showing F_IS_ < −0.5 and F_ST_ > 0.3 in the Tyrrhenian population.

The analysis of single F_IS_ and F_ST_ values allowed us to identify several variants potentially associated with SD, according to the criteria established (F_IS_ < −0.5 and F_ST_ > 0.3). Only five variants met those criteria for the Aegean population (Figure 3), while nine variants were found for the Tyrrhenian population (Figure 4). All the candidate variants were in different contigs and positions in both populations, except for an intron of SEC14 and spectrin domains 1 (sestd1) gene from the scaffold_2829 p412770 shared by both geographical regions (Table S7).

### 3.7. SD Candidate Variants

Of the five sex-associated variants detected in the Aegean region, four were intergenic and one was intronic. The association of genotypes with sex-adjusted either to a ZW/ZZ system for three SNPs or to a XX/XY system for another two SNPs (Table 1; Figure 3). Focusing on the four intergenic variants, two were consistent with an XY sex-determining system, where the Y-allele frequency in males ranged between 0.375 and 0.400. Conversely, the other two intergenic variants were consistent with a ZW SD system, where the W-allele frequency in females ranged from 0.370 and 0.43.

In the Tyrrhenian population, three analyses with different datasets were performed. A first analysis was initially performed on a subset of samples that reflected natural frequencies. The frequencies of males with the expected fshr genotype (WT/mut1), males with a non-concordant genotype (WT/WT), females with the expected fshr genotype (WT/WT), and females with a non-concordant genotype (WT/mut1) were balanced based on the work reported in Ferraresso et al. [18]. Despite the small sample size, one fshr variant showed a significant association with phenotypic sex, confirming the results reported in Ferraresso et al. [18]. Moreover, other loci showing possible sex-associated were detected.

A second comparison was carried out using only males with a non-concordant genotype (WT/WT) versus females with the expected fshr genotype (WT/WT)). In this case, several variants, but especially the intronic variant on the sestd1 gene, were found.

In the third comparison, all available individuals were used, 12 females with the expected WT/WT fshr genotype, 4 females with genotype WT/mut1, 11 males were with the WT/mut1 genotype, and 9 males with the genotype WT/WT. Only nine variants with a sex-association were found, and these included the aforementioned intronic variant on sestd1 (Table 2; Figure 4).

After identifying the sex-associated SNPs, the flanking regions within ±100 kb of each variant were analysed, several genes were detected within these intervals. Only a few flanking genes could be linked to biological pathways or processes related to sex determination (Supplementary Materials, Tables S2 and S3) [62,63,64,65,66,67,68,69,70,71,72,73,74,75,76].

## 4. Discussion

The mechanism underlying SD in teleosts is known to depend on different genetic and environmental factors. Several studies have reported the presence of male heterogamety (XY system), female heterogamety (ZW system), or polygenic SD with or without multiple sex chromosomes [12,16]. SD might also show intraspecific variation [22]. Various environmental factors might be involved by influencing, partially or totally, the gonad differentiation cascade at the top or bottom level in different stages of growth [15]. Genetic and environmental factors may also interact to determine phenotypic sex. For instance, in the pejerrey (*Odontesthes bonariensis*), *amhy* has been consistently identified as a MSD gene [77]. However, in this species, SD is also strongly influenced by temperature. If the temperature during a critical stage is around 17 °C, a 100% female sex ratio is observed. Conversely, if the temperature is around 29 °C, a 100% male sex ratio is achieved [78]. Another well documented case is the European sea bass (*Dicentrarchus labrax*), where early exposure to low temperature (16 °C) increases the proportion of females, but prolonged exposure leads to a sex ratio skewed towards males by up to 90% [79,80]

The flathead grey mullet apparently shows a complex mechanism of SD with several of the features summarised above. The present study builds upon the work of Ferraresso et al. [18], utilising whole-genome re-sequencing on individual samples and extending the analysis on the Aegean population that was previously analysed only with a targeted locus approach. Single-individual WGS data were analysed against a novel and more complete genome reference, allowing for greater accuracy in SNP calling and mapping and a much higher resolution genome scan to identify potential sex-linked variants. Although the *fshr* gene has previously been reported to be the putative MSD gene in *M. cephalus*, not all individuals showed the expected correspondence between genotype and phenotype [18,19,21]. Despite the limited sample size, the individual whole-genome re-sequencing approach confirmed the potential role of *fshr* in the Tyrrhenian population with the natural frequencies. Likewise, the already known variants at this locus showed no significant association with sex in the Aegean population, as already reported in [18]. Extending the analysis to other SNPs within the *fshr* region clearly showed that this locus is not significantly involved in sex determination in the Aegean population sample. For this population, five SNPs, located on different contigs, all in non-coding regions, showed a significant association with phenotypic sex. The most interesting one is the intronic variant located within the *sestd1* gene. The encoded protein has been shown to interact with key components of the β-catenin-dependent and independent Wnt signalling pathways [81,82], which is known to have an important role in sex determination and gonadal development in vertebrates. The finding that putative SD SNPs are located in non-coding regions, either in introns or intergenic regions, at variance with the evidence for *fshr* might be explained with the hypothesis that they are involved the regulation of gene expression. It is well documented that intronic variants may play a relevant role in mRNA transcription [83,84,85,86]. They can activate cryptic splice sites (CSS), resulting in the inclusion of intronic sequence or the partial exclusion of exons in the mature mRNA [87], leading to an altered protein with possible effects on its function [88,89]. Moreover, many enhancers are located within introns and intergenic regions [90,91]. Understanding the effects of these non-coding variants and identifying their target gene represents a significant challenge [88].

Considering the limited sample size, these putative SD loci remain to be fully validated on a larger set of animals. However, it is already evident, even with the current sample size, that all putative SD variants in the Aegean population showed only incomplete association with phenotypic sex, therefore it seems that in this population there is not an equivalent of the *fshr* locus and an MSD may not exist at all.

The evidence of incomplete association between any identified genetic variants and phenotypic sex points toward not only the absence of an MSD for the flathead grey mullet, but also to the possible role of multiple loci that differ across populations. A polygenic genetic architecture has been already proposed for other species such as the European sea bass [92], and the same species was reported to show variation in GSD across populations [93]. A second, not mutually exclusive hypothesis is that environmental factors might reduce the effect of genetic ones. It should also be considered that genetic variants might determine the response to environmental variables as in the case of temperature for the European sea bass [94].

In the case of *M. cephalus*, the environmental component might explain the incomplete concordance between *fshr* genotypes and phenotypic sex observed in the Tyrrhenian and, the absence of putative MSD in the Aegean. In the European sea bass and the tongue sole (*Cynoglossus semilaevis*), temperature can modify the final sex via epigenetic regulation and/or modulating the stress hormone-mediated pathway [95,96]. It is tempting to speculate that temperature could play a role in mullet SD as well, which might explain the differences observed between the Aegean and the Tyrrhenian populations. The Aegean Sea is typically warmer than the Tyrrhenian Sea and a long-term trend analysis suggests that the eastern Mediterranean is experiencing faster warming rates compared to the western part [97,98,99].

## 5. Conclusions

While the individual WGS allowed a genome scan for SD variants at single nucleotide resolution, the current sample size was limited and the putative SD loci remain to be fully confirmed. On the other hand, even with the highest possible resolution at the whole genome level there was no evidence for an MSD locus, especially for the Aegean population. This suggests that either several genetic loci contribute to SD in the grey mullet in a population-specific manner or local environmental factors are at play. As mentioned, these two hypotheses are not mutually exclusive, but both require a much more challenging experimental design to be fully tested. In the first case, a genome-wide association study (GWAS) on a much larger experimental population raised in the same controlled environment would be necessary for the GWAS to reliably identify major and minor loci that contribute to phenotypic sex and to assess the relative contribution. To perform a robust GWAS, it is necessary to produce a large number of families under controlled conditions, using parents from each population under study, if differences between populations are the objective. In the case of environmental factors, hatchery-produced mullets need to be raised from the early larval stages under controlled conditions testing the influence of specific external variables (e.g., high or low temperatures) on sex ratios in a trial-and-error process. Both approaches are currently quite challenging as artificial reproduction in the grey mullet is not as advanced as in other well-studied species, such as the European sea bass.

Finally, considering the complexity of SD in the flathead grey mullet and in fish in general, it would be interesting to study SD in other mullet species of the Mugilidae family, as has already been done in other teleost taxonomic groups such as flatfishes [20].

## Figures and Tables

**Table 1:** Main features of SNPs associated with sex in the Aegean population. AN (total number of alleles called), AC (allele count in genotypes), AF (allele frequency), MDP (mean read depth) and NA (not available).

Aegean Population			Female			Male							
CIBA_Chr.	Contig	Pos. in bp	AN	AC	AF	AN	AC	AF	System	Location	Gene	MDP	*p*-Value
18	contig_3267	427998	32	0	0	40	16	0.4	XY	Intergenic	NA	26.78	3.2 × 10^−5^
16	contig_761	3728	32	14	0.4375	40	2	0.05	ZW	Intergenic	NA	187.92	6.2 × 10^−5^
19	contig_948	1665937	32	0	0	40	15	0.375	XY	Intergenic	NA	24.75	3 × 10^−5^
12	scaffold_2829	4121770	32	11	0.34375	40	0	0	ZW	Intronic	*sestd1*	21.14	4.6 × 10^−5^
9	scaffold_520	15101365	32	12	0.375	40	1	0.025	ZW	Intergenic	NA	25.69	0.000342

**Table 2:** Main features of SNPs associated with sex in the Tyrrhenian population. AN (total number of alleles called), AC (allele count in genotypes), AF (allele frequency), MDP (mean read depth) and NA (not available).

Tyrrhenian Population			Female			Male							
CIBA_Chr.	Contig	Pos. in bp	AN	AC	AF	AN	AC	AF	System	Location	Gene	MDP	*p*-Value
12	contig_1367	584196	32	0	0	40	15	0.375	XY	Intronic	*kalrn_2*	14.39	5.2 × 10^−5^
11	contig_2266	559	32	0	0	40	15	0.375	XY	Intergenic	NA	182.31	2 × 10^−5^
21	contig_2858	378720	32	12	0.375	40	1	0.025	ZW	Intergenic	NA	6.28	0.000288
3	contig_434	4708990	32	11	0.344	40	0	0	ZW	Intergenic	NA	19	4 × 10^−6^
16	contig_572	473849	32	13	0.406	40	2	0.05	ZW	Intronic	*mpp3*	24.61	0.000268
8	contig_892	4477096	32	14	0.438	40	0	0	ZW	Intronic	*gnaq_2*	20.61	0
19	contig_948	5446550	32	0	0	40	15	0.375	XY	Intergenic	NA	11.58	1.4 × 10^−5^
5	scaffold_2265	10476908	32	12	0.375	40	1	0.25	ZW	Intronic	*limch1_2*	16.69	0.000304
12	scaffold_2829	4121770	32	11	0.344	40	0	0	ZW	Intronic	*sestd1*	20.08	2.4 × 10^−5^

## Data Availability

In present study, all the data were deposited in the two NCBI BioProject, accession number PRJNA1221841 for Illumina data and accession number PRJNA1220361 for genome data.

## Supplementary Materials

The following supporting information can be downloaded at: https://www.mdpi.com/article/10.3390/ani15162445/s1. Table S1 Chromosome associations between the CIBA_Mcephalus_1.1 reference genome and the reduced assembled genome; Table S2 Genotype distribution of fshr in 72 individuals (32 females, 40 males) from two geographic regions; Table S3 Transposable element results; Table S4 Summary of Illumina sequencing read counts; Table S5 Comparative report of genome contiguity and completeness; Table S6 Number of SNPs per chromosome in each population; Table S7 Summary of candidate variants potentially linked to SD across populations; Figure S1 Location of mitochondrial protein genes M. cephalus mitochondrial genome; Figure S2 Scatterplot of DAPC analysis for Tyrrhenian population using 2,835,032 SNPs; Table S8 Metrics results from BUSCO and QUAST results for the genome assembly; Table S9 Intergenic variants identified in the Aegean population and annotation of genes present within ±100 kb of each variant’s position. NA (not available). Table S10 Intergenic variants identified in the Tyrrhenian population and annotation of genes present within ±100 kb of each variant’s position. NA (not available).
