# Supplementary material for: Complex Sex Determination in the Grey Mullet Mugil cephalus Suggested by Individual Whole Genome Sequence Data
Source: Animals (Basel). 2025 Aug 20;15(16):2445. doi: 10.3390/ani15162445 (PMC12382950; doi:10.3390/ani15162445)
Supplement: Supplementary file 1 [file animals-15-02445-s001.zip › Glossary.pdf]

Sex determination (SD)

follicle-stimulating hormone receptor gene (*fshr*)

Master Sex determination (MSD)

genetic sex determination (GSD)

environmental sex determination (ESD)

single-nucleotide polymorphisms (SNP)

transposable element (TE)

whole genome sequencing (WGS)

minor allele frequency (MAF)

Discriminant Analysis of Principal Components (DAPC)

Linkage disequilibrium (LD)

Oxford Nanopore Technologies (ONT)

pair-end (PE)

base pair bp

chromosome (C)

SEC14 and spectrin domains 1 (*sestd1)*

total number of alleles in called (AN)

allele count in genotypes (AC)

allele frequency (AF)

MAGUK p55 subfamily member 3 (*mpp3*)

Guanine nucleotide-binding protein G(q) subunit alpha (*gnaq_2*)

LIM and calponin homology domains-containing protein 1 gene (*limch1_2*)

activin receptor type-2B (*acvr2b*)

tartrate-resistant acid phosphatase type 5 (*acp5*)

phosphodiesterase 1C (*pde1c*)

phosphodiesterase 9A (*pde9a*),

5-hydroxytryptamine receptor 3A (*htr3a*)

rabphilin 3A (*rph3a*)

tyrosine-protein phosphatase non-receptor type 11(*ptpn11*)

DEAD-Box Helicase 55(*ddxX55*)

Days post fertilization (dpf)

prostaglandin E2 receptor EP4 subtype b (*ptger4*)

sperm tail PG-rich repeat-containing protein 2 (*stpg2*)

forkhead box B2 (*foxb2*)

Planar Cell Polarity (PCP)

sex steroid-responsive peptidergic (FeSP)

cryptic splice sites (CSS)
