# Supplementary material for: Complex Sex Determination in the Grey Mullet Mugil cephalus Suggested by Individual Whole Genome Sequence Data
Source: Animals (Basel). 2025 Aug 20;15(16):2445. doi: 10.3390/ani15162445 (PMC12382950; doi:10.3390/ani15162445)
Supplement: Supplementary file 1 [file animals-15-02445-s001.zip › Supplementary Figures S1-S2; Tables S8-S10.pdf]

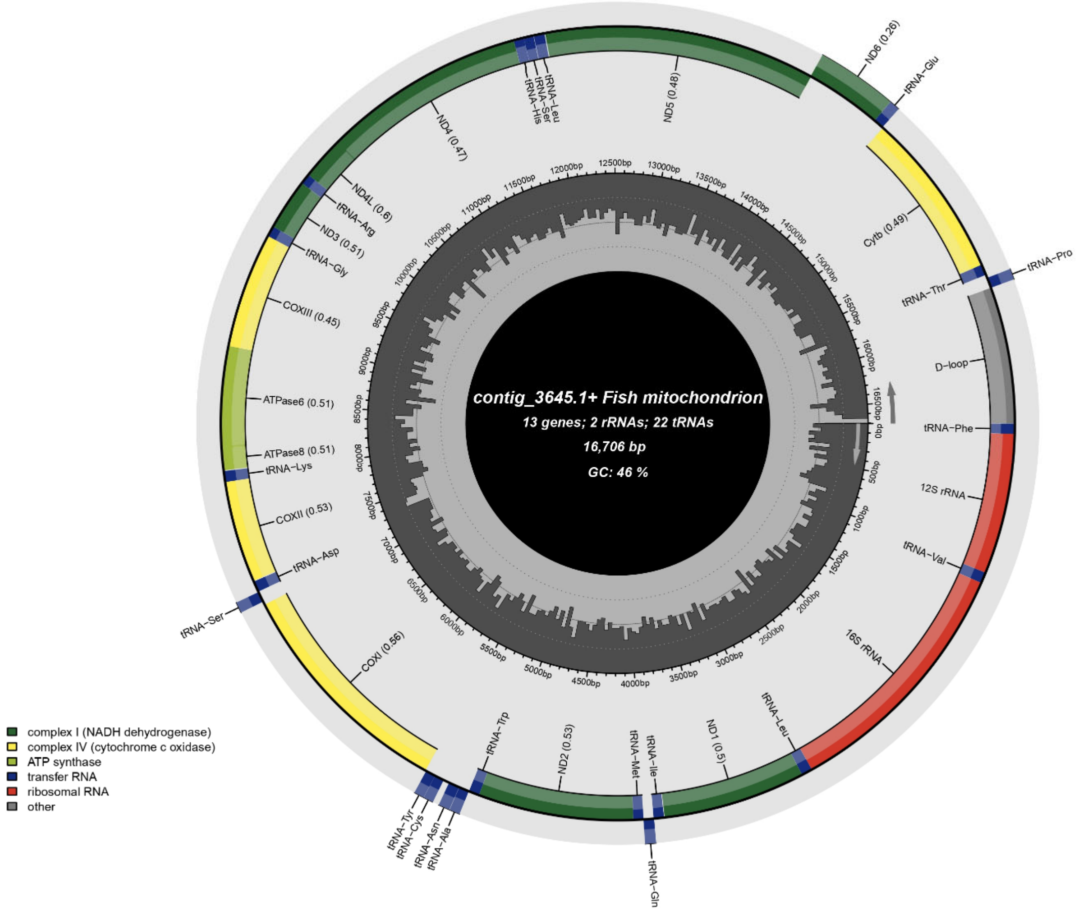

Figure S1 Location of mitochondrial protein genes *M. cephalus* mitochondrial genome.

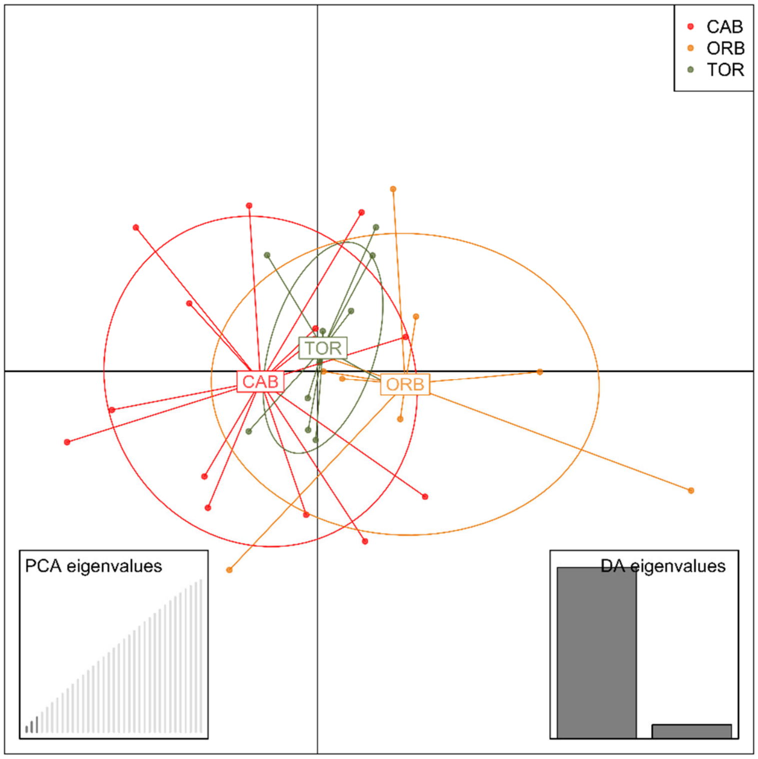

Figure S2 Scatterplot of DAPC analysis for Tyrrhenian population using 2,835,032 SNPs.

QUAST	
Assembly	Mcephalus_assembly
# contigs (>= 0 bp)	1304
# contigs (>= 1000 bp)	1266
# contigs (>= 5000 bp)	973
# contigs (>= 10000 bp)	797
# contigs (>= 25000 bp)	619
# contigs (>= 50000 bp)	474
Total length (>= 0 bp)	658524508
Total length (>= 1000 bp)	658500290
Total length (>= 5000 bp)	657650484
Total length (>= 10000 bp)	656383864
Total length (>= 25000 bp)	653545798
Total length (>= 50000 bp)	648251801
# contigs	1295
Largest contig	30465394
Total length	658521108
GC (%)	41.85
N50	8379386
N75	3415910
L50	21
L75	52
# N's per 100 kbp	0

BUSCO	
C:98.2%[S:97.6%,D:0.6%],F:0.4%,M:1.4%,n:3640	
3573	Complete BUSCOs ©
3551	Complete and single-copy BUSCOs (S)
22	Complete and duplicated BUSCOs (D)
16	Fragmented BUSCOs (F)
51	Missing BUSCOs (M)
26	Total BUSCO groups searched

Table S8. Metrics results from BUSCO and QUAST results for the genome assembly.

Aegean population						
**Contig**	**Position in bp**	**Variant type**	**Gene location**	**gene detected in the ±100kb regions**	**full name**	**Ref**
contig_948	1665937	Intergenic	NA	rph3a_2	Rabphilin-3A	[65]
				ptpn11	Tyrosine-protein phosphatase non-receptor type 11	[66]
				ddx55	ATP-dependent RNA helicase DDX55	[67,68]
scaffold_520	15101365	Intergenic	NA	pde9a_2	High affinity cGMP-specific 3'%2C5'-cyclic phosphodiestera	[63]
contig_3267	427998	Intergenic	NA	acvr2b	Activin receptor type-2B	[61]
				acp5_2	Tartrate-resistant acid phosphatase type 5	[61]
				pde1c_3	Calcium/calmodulin-dependent 3'%2C5'-cyclic nucleotide phosphodiesterase 1C	[62]
				pde1c_4	Calcium/calmodulin-dependent 3'%2C5'-cyclic nucleotide phosphodiesterase 1C	[62]
contig_761	3728	Intergenic	NA	htr3b_2	5-hydroxytryptamine receptor 3B	[64]

Table S9 Intergenic variants identified in the Aegean population and annotation of genes present within ±100 kb of each variant's position. NA (not available).

Tyrrhenian population						
**Contig**	**Position in bp**	**Variant type**	**Gene location**	**gene detected in the ±100kb regions**	**full name**	**Ref**
contig_2266	559	Intergenic	NA	NA	NA	
contig_434	4708990	Intergenic	NA	NA	NA	
contig_2858	378720	Intergenic	NA	NA	NA	
scaffold_2265	10476908	intronic	limch1_2	NA	NA	
contig_948	5446550	Intergenic	NA	ptger4_3	Prostaglandin E2 receptor EP4 subtype	[69]
contig_892	4477096	intronic	gnaq_2	stpg2	Sperm-tail PG-rich repeat-containing protein 2	[71]
				stpg2_2	Sperm-tail PG-rich repeat-containing protein 2	[71]
				gnaq_3	Guanine nucleotide-binding protein G(q) subunit alpha	[70]
				foxb2	Forkhead box protein B2	[72]
contig_572	473849	intronic	mpp3 [74]	lrp2_2	Low-density lipoprotein receptor-related protein 2	[75]
contig_1367	584196	intronic	kalrn_2	kalrn_6	Kalirin	[73]
				kalrn_4	Kalirin	[73]

Table S10 Intergenic variants identified in the Tyrrhenian population and annotation of genes present within ±100 kb of each variant's position. NA (not available).

61. Zhang, Y.; Cao, X.; Zou, Y.; Yan, Z.; Huang, Y.; Zhu, Y.; Gao, J. De Novo Gonad Transcriptome Analysis of Elongate Loach (*Leptobotia Elongata*) Provides Novel Insights into Sex-Related Genes. *Comp. Biochem. Physiol. Part D Genomics Proteomics* **2022**, *42*, 100962, doi:10.1016/j.cbd.2022.100962.
62. Meng, F.; Sun, S.; Xu, X.; Yu, W.; Gan, R.; Zhang, L.; Zhang, W. Transcriptomic Analysis Provides Insights into the Growth and Maturation of Ovarian Follicles in the Ricefield Eel (*Monopterus Albus*). *Aquaculture* **2022**, *555*, 738251, doi:10.1016/j.aquaculture.2022.738251.

63. Li, J.; Bai, L.; Liu, Z.; Wang, W. Dual Roles of PDE9a in Meiotic Maturation of Zebrafish Oocytes. *Biochem. Biophys. Res. Commun.* **2020**, *532*, 40–46, doi:10.1016/j.bbrc.2020.08.005.
64. Evsiukova, V.S.; Kulikova, E.A.; Kulikov, A.V. Age-Related Alterations in the Behavior and Serotonin-Related Gene mRNA Levels in the Brain of Males and Females of Short-Lived Turquoise Killifish (Nothobranchius Furzeri). *Biomolecules* **2021**, *11*, 1421, doi:10.3390/biom11101421.
65. Qu, J.; Li, R.; Xie, Y.; Liu, Y.; Liu, J.; Zhang, Q. Differential Transcriptomic Profiling Provides New Insights into Oocyte Development and Lipid Droplet Formation in Japanese Flounder (*Paralichthys Olivaceus*). *Aquaculture* **2022**, *550*, 737843, doi:10.1016/j.aquaculture.2021.737843.
66. He, Z.; Deng, F.; Yang, D.; He, Z.; Hu, J.; Ma, Z.; Zhang, Q.; He, J.; Ye, L.; Chen, H.; et al. Crosstalk between Sex-Related Genes and Apoptosis Signaling Reveals Molecular Insights into Sex Change in a Protogynous Hermaphroditic Teleost Fish, Ricefield Eel *Monopterus Albus*. *Aquaculture* **2022**, *552*, 737918, doi:10.1016/j.aquaculture.2022.737918.
67. He, L.; Wang, Q.; Jin, X.; Wang, Y.; Chen, L.; Liu, L.; Wang, Y. Transcriptome Profiling of Testis during Sexual Maturation Stages in Eriocheir Sinensis Using Illumina Sequencing. *PLOS ONE* **2012**, *7*, e33735, doi:10.1371/journal.pone.0033735.
68. Pauletto, M.; Milan, M.; Huvet, A.; Corporeau, C.; Suquet, M.; Planas, J.V.; Moreira, R.; Figueras, A.; Novoa, B.; Patarnello, T.; et al. Transcriptomic Features of Pecten Maximus Oocyte Quality and Maturation. *PLOS ONE* **2017**, *12*, e0172805, doi:10.1371/journal.pone.0172805.
69. Sreenivasan, R.; Jiang, J.; Wang, X.; Bártfai, R.; Kwan, H.Y.; Christoffels, A.; Orbán, L. Gonad Differentiation in Zebrafish Is Regulated by the Canonical Wnt Signaling Pathway1. *Biol. Reprod.* **2014**, *90*, 45, 1–10, doi:10.1095/biolreprod.113.110874.
70. Wang, C.; Yang, L.; Xiao, T.; Li, J.; Liu, Q.; Xiong, S. Identification and Expression Analysis of Zebrafish Gnaq in the Hypothalamic–Pituitary–Gonadal Axis. *Front. Genet.* **2022**, *13*, doi:10.3389/fgene.2022.1015796.
71. Kubo, S.; Black, C.S.; Joachimiak, E.; Yang, S.K.; Legal, T.; Peri, K.; Khalifa, A.A.Z.; Ghanaeian, A.; McCafferty, C.L.; Valente-Paterno, M.; et al. Native Doublet Microtubules from Tetrahymena Thermophila Reveal the Importance of Outer Junction Proteins. *Nat. Commun.* **2023**, *14*, 2168, doi:10.1038/s41467-023-37868-0.
72. Hu, L.; Chen, W.; Qian, A.; Li, Y.-P. Wnt/β-Catenin Signaling Components and Mechanisms in Bone Formation, Homeostasis, and Disease. Bone Res. 2024, 12, 1–33, doi:10.1038/s41413-024-00342-8.
73. Kratzer, M.-C.; England, L.; Apel, D.; Hassel, M.; Borchers, A. Evolution of the Rho Guanine Nucleotide Exchange Factors Kalirin and Trio and Their Gene Expression in Xenopus Development. Gene Expr. Patterns 2019, 32, 18–27, doi:10.1016/j.gep.2019.02.004.
74. Beal, A.P.; Martin, F.D.; Hale, M.C. Using RNA-Seq to Determine Patterns of Sex-Bias in Gene Expression in the Brain of the Sex-Role Reversed Gulf Pipefish (Syngnathus Scovelli). Mar. Genomics 2018, 37, 120–127, doi:10.1016/j.margen.2017.09.005.
75. Wang, M.; Chen, L.; Zhou, Z.; Xiao, J.; Chen, B.; Huang, P.; Li, C.; Xue, Y.; Liu, R.; Bai, Y.; et al. Comparative Transcriptome Analysis of Early Sexual Differentiation in the Male and Female Gonads of Common Carp (Cyprinus Carpio). Aquaculture 2023, 563, 738984, doi:10.1016/j.aquaculture.2022.738984.
